# Detection of Subclinical Diabetic Neuropathy in Type 2 Diabetes: A Study of Nerve Conduction Parameters and Their Associations With Metabolic and Demographic Factors

**DOI:** 10.7759/cureus.100625

**Published:** 2026-01-02

**Authors:** Suyash Litoriya, Umar Khan, Sahebrao Kishanrao Sadawarte

**Affiliations:** 1 Internal Medicine, People's College of Medical Sciences & Research Centre, Bhopal, IND; 2 Physiology, People's College of Medical Sciences & Research Centre, Bhopal, IND

**Keywords:** f-wave, hba1c, nerve conduction study (ncs), subclinical diabetic neuropathy, type 2 diabetes mellitus

## Abstract

Background

Diabetic peripheral neuropathy (DPN) is one of the most common and disabling complications of type 2 diabetes mellitus (T2DM), affecting a substantial proportion of patients and contributing to significant morbidity and reduced quality of life. Importantly, up to one-third of individuals may already have subclinical electrophysiological abnormalities at the time of diagnosis, highlighting the need for early detection before symptoms become clinically evident. A considerable proportion of patients develop nerve conduction abnormalities even before neuropathic symptoms appear on clinical examination. Nerve conduction studies (NCS), including F-wave assessment, offer a sensitive approach to detecting early, subclinical nerve dysfunction. This study evaluated detailed NCS parameters in neurologically asymptomatic T2DM patients and examined their associations with key metabolic and demographic factors.

Methods

A prospective, cross-sectional, observational study was conducted involving 60 participants aged 18-70 years, comprising 30 neurologically asymptomatic individuals with T2DM and 30 age- and sex-matched healthy controls. Neurologically asymptomatic status was confirmed through a structured clinical neurological examination and screening using the Michigan Neuropathy Screening Instrument (MNSI), with participants excluded if they demonstrated clinical signs or scored above the diagnostic threshold for neuropathy. Standardized exclusion criteria were applied to minimize confounding, including known vitamin B12 deficiency, chronic alcohol use, renal insufficiency, thyroid dysfunction, exposure to neurotoxic medications, or other neurological disorders. Motor and sensory NCS were performed on upper- and lower-limb nerves to assess distal latencies, conduction velocities, compound muscle action potential (CMAP) amplitudes, sensory nerve action potential (SNAP) amplitudes, and F-wave minimum latencies. A sample size of 60 was determined based on feasibility and prior reports, acknowledging that no formal power calculation was performed and recognizing this as a limitation. Statistical analysis included independent Student’s t-tests for between-group comparisons, and Pearson’s correlation to explore associations with glycated hemoglobin (HbA1c), age, duration of diabetes, and body mass index (BMI), following verification of approximate normal distribution of continuous variables.

Results

Compared with healthy controls, participants with T2DM demonstrated significant impairments across the majority of NCS parameters (predominantly p < 0.001), characterized by prolonged distal and F-wave latencies, reduced CMAP and SNAP amplitudes, and slower conduction velocities in both upper- and lower-limb nerves. Correlation analysis showed weak-to-moderate associations between HbA1c, age, and duration of diabetes with key conduction indices (r values generally ranging from ±0.30 to ±0.75, p < 0.05), while BMI showed no meaningful correlation with NCS measures.

Conclusion

Neurologically asymptomatic T2DM patients showed early electrophysiological changes suggestive of subclinical DPN. NCS, including F-wave assessment, may aid early identification, though our cross-sectional design and small sample size limit causal conclusions regarding prevention or long-term outcomes.

## Introduction

Type 2 diabetes mellitus (T2DM) is a highly prevalent, chronic metabolic disease and a major cause of long-term morbidity and mortality worldwide. Persistent hyperglycemia contributes to microvascular and macrovascular complications. Among these, diabetic peripheral neuropathy (DPN) is one of the most frequent and disabling, affecting up to 50% of individuals with longstanding T2DM and leading to pain, sensory loss, gait disturbance, ulceration, and infection. Its association with non-traumatic lower-limb amputation imposes a considerable economic and quality-of-life burden on patients and health-care systems [1-9].

Diabetic neuropathy is defined as a peripheral nerve disorder attributable to diabetes mellitus, after reasonable exclusion of alternative etiologies, and may be clinically apparent or remain subclinical [5-9]. The most common form is a length-dependent, symmetrical, predominantly sensory polyneuropathy that typically begins distally in the lower limbs. Although many individuals eventually develop paresthesia, burning pain, and numbness in a stocking distribution, electrophysiological abnormalities may develop early in the disease course, even when patients are neurologically asymptomatic on clinical examination, highlighting an important opportunity for early detection and intervention [5-9].

Nerve conduction studies (NCS) are a key electrodiagnostic method for evaluating peripheral nerve function in diabetes, assessing distal latencies, compound muscle action potential (CMAP) and sensory nerve action potential (SNAP) amplitudes, and motor conduction velocity (MCV) and sensory conduction velocity (SCV) [10-12]. F-wave responses, which traverse longer proximal motor pathways, may detect abnormalities earlier than conventional distal parameters, making them useful in identifying subclinical involvement [10-12].

Glycemic status is a key determinant of peripheral nerve integrity, with chronic hyperglycemia contributing to metabolic and microvascular injury. Higher glycated hemoglobin (HbA1c) levels, increasing age, and longer duration of diabetes have been consistently linked with more pronounced nerve conduction abnormalities, whereas evidence regarding the independent contribution of body mass index (BMI) to early neurophysiological changes remains heterogeneous and inconclusive [13-19].

The present study aimed to evaluate motor and sensory nerve conduction parameters, including F-wave minimum latencies, in neurologically asymptomatic patients with T2DM in comparison with healthy controls, and to assess their correlations with HbA1c, age, duration of diabetes, and BMI, to better understand their relative impact on subclinical nerve dysfunction.

## Materials and methods

Study design and setting

This was a prospective, cross-sectional study conducted from August to October 2022 in the Outpatient Department of People’s Hospital, affiliated with People’s College of Medical Sciences and Research Centre (PCMS & RC), Bhopal, India. The Institutional Ethics Committee (IEC) of PCMS & RC issued approval for this study (approval no. ECR/519/Inst/MP/2014/RR-2020; dated August 29, 2022).

Participants and study groups

A total of 60 participants, aged 18-70 years, were enrolled and allocated equally into two groups (30 with T2DM and 30 healthy controls). The diabetic group comprised neurologically asymptomatic patients with T2DM, defined by a normal structured neurological examination and Michigan Neuropathy Screening Instrument (MNSI) assessment [20], with individuals exceeding the diagnostic threshold excluded. The control group included apparently healthy, non-diabetic individuals. Both males and females were included. Institutional ethics approval was obtained prior to study initiation, and written informed consent was obtained from all participants.

Inclusion and exclusion criteria

Participants in both groups were aged 18-70 years. The diabetic group had a confirmed diagnosis of T2DM for ≤10 years. Controls had no history of diabetes and normal glycemic indices (fasting plasma glucose <126 mg/dL, two-hour post-prandial glucose <200 mg/dL, and HbA1c <6.5%). Individuals with potential confounders (e.g., B12 deficiency, renal disease, thyroid dysfunction, chronic alcohol use, neurotoxic medications, or other neurological disorders) were excluded.

Participants were excluded if they had established clinical diabetic neuropathy or other neurological illnesses that could affect peripheral nerves, such as radiculopathy, plexopathy, or hereditary neuropathy. Individuals with chronic kidney disease, thyroid dysfunction, or severe anemia were also excluded. Further exclusion criteria were chronic alcoholism, current smoking, significant occupational exposure to neurotoxic heavy metals, or the use of medications known to affect nerve conduction, including phenytoin, certain diuretics, or high-dose vitamin B12 or B6 supplementation.

Clinical and laboratory data collection

For each subject, age, gender, duration of diabetes, age at onset of diabetes (for the diabetic group), height, weight, BMI (kg/m²), and blood pressure were recorded using a structured proforma. HbA1c values obtained within the preceding 15 days were used to represent glycemic control.

Nerve conduction studies (NCS)

All NCS were performed using the NEURO PERFECT EMG-200 EMG/NCV/EP system (Medicaid Systems, Chandigarh, India). Examinations were carried out in a quiet room, with ambient temperature maintained between 26°C and 30°C, and skin temperature at the recording sites was ensured to be above 32°C. Standard surface electrodes and supramaximal percutaneous stimulation were used, and all recordings were obtained from the right side to maintain uniformity.

In the upper limb, motor and sensory components of the median, ulnar, and radial nerves were evaluated. In the lower limb, motor studies were performed on the common peroneal and posterior tibial nerves, and sensory studies were performed on the sural nerve. For motor studies, distal motor latency (DML), CMAP amplitude, and MCV were measured, and F-wave minimum latency was obtained for each motor nerve. For sensory studies, distal sensory latency (DSL), SNAP amplitude, and SCV were measured.

Sample size and treatment profile

The final sample comprised 60 subjects, with 30 participants in each group. Overall, 36 (60.0%) were males, and 24 (40.0%) were females, giving a male-to-female ratio of 1.5:1. Among the 30 diabetic patients, 25 (83.3%) were receiving oral antidiabetic agents, and five (16.7%) were on insulin therapy at the time of assessment.

Statistical analysis

Data were entered and analyzed using IBM SPSS Statistics for Windows, Version 20 (Released 2011; IBM Corp., Armonk, NY, USA). Continuous variables were expressed as mean ± standard deviation (SD). Between-group comparisons of NCS parameters (diabetic vs. control) were performed using the independent Student’s t-test. Associations between NCS parameters and HbA1c, age, duration of diabetes, and BMI among diabetic participants were assessed using Karl Pearson’s correlation coefficient, and the corresponding t statistic was derived for each correlation coefficient, with degrees of freedom (df) = n - 2. A p-value ≤0.05 was considered statistically significant. Sample sizes are reported in the format n (%).

## Results

Baseline characteristics

The 60 study participants included 36 (60.0%) males and 24 (40.0%) females. The mean age of the diabetic group was 48.23 ± 12.07 years, and the mean age of the control group was 42.84 ± 12.14 years. Among the diabetic participants, the mean age at onset of diabetes was 45.81 ± 11.35 years, the mean BMI was 25.05 ± 2.09 kg/m², and the mean duration of diabetes was 3.38 ± 2.29 years.

Of the 30 diabetic patients, 25 (83.3%) were on oral antidiabetic drugs, and five (16.7%) were receiving insulin.

Comparison of upper-limb NCS parameters between diabetics and controls

Diabetic patients showed markedly prolonged DML, DSL, and F-wave minimum latencies, along with significantly reduced CMAP and SNAP amplitudes, and slower MCV and SCV in the median, radial, and ulnar nerves, compared with controls. All differences were highly significant (p < 0.001), with large t values supporting robust group separation (Table 1).

**Table 1:** Comparison of various motor and sensory NCS parameters of upper limb between diabetic patients and controls Independent Student’s t-test; df = 58 for all comparisons NCS: nerve conduction studies; CMAP: compound muscle action potential; SNAP: sensory nerve action potential; HS: highly significant (p < 0.001)

Parameter	Nerve	Diabetics (n = 30), mean ± SD	Controls (n = 30), mean ± SD	Test statistic (t)	p-value	Significance
Distal Motor Latency (ms)	Median	7.54 ± 1.51	3.23 ± 1.19	12.28	<0.001	HS
	Radial	7.56 ± 1.42	3.67 ± 1.25	11.26	<0.001	HS
	Ulnar	7.35 ± 1.48	3.83 ± 1.13	10.35	<0.001	HS
CMAP Amplitude (mV)	Median	3.24 ± 1.22	7.49 ± 1.26	13.27	<0.001	HS
	Radial	3.78 ± 1.23	7.82 ± 1.22	12.77	<0.001	HS
	Ulnar	3.07 ± 1.18	7.16 ± 1.28	12.87	<0.001	HS
Motor Conduction Velocity (m/s)	Median	31.41 ± 4.82	51.86 ± 5.12	15.93	<0.001	HS
	Radial	32.02 ± 4.51	50.24 ± 4.19	16.21	<0.001	HS
	Ulnar	32.65 ± 4.77	52.02 ± 5.15	15.11	<0.001	HS
F-wave Minimum Latency (ms)	Median	37.31 ± 3.33	26.72 ± 1.82	15.28	<0.001	HS
	Radial	37.47 ± 3.38	26.48 ± 1.79	15.74	<0.001	HS
	Ulnar	37.33 ± 3.31	26.64 ± 1.27	16.52	<0.001	HS
Distal Sensory Latency (ms)	Median	7.90 ± 1.90	3.23 ± 1.29	11.14	<0.001	HS
	Radial	7.92 ± 1.89	3.92 ± 1.59	8.87	<0.001	HS
	Ulnar	7.93 ± 1.77	3.13 ± 1.72	10.65	<0.001	HS
SNAP Amplitude (µV)	Median	5.24 ± 2.04	8.22 ± 1.72	6.12	<0.001	HS
	Radial	5.01 ± 2.02	8.73 ± 2.28	6.69	<0.001	HS
	Ulnar	5.16 ± 1.88	8.18 ± 2.34	5.51	<0.001	HS
Sensory Conduction Velocity (m/s)	Median	33.37 ± 3.42	47.89 ± 3.82	15.51	<0.001	HS
	Radial	33.76 ± 3.57	47.46 ± 3.14	15.78	<0.001	HS
	Ulnar	33.32 ± 3.63	47.03 ± 3.20	15.52	<0.001	HS

Comparison of lower-limb NCS parameters between diabetics and controls

In the common peroneal and posterior tibial motor nerves, diabetics demonstrated significantly longer DMLs, lower CMAP amplitudes, and slower conduction velocities than controls. For the sural sensory nerve, DSL was markedly prolonged, SNAP amplitude was reduced, and SCV was substantially slowed in diabetic patients. All differences were highly significant (p < 0.001), confirming consistent electrophysiological evidence of subclinical neuropathy affecting both upper- and lower-limb nerves in neurologically asymptomatic T2DM patients (Table 2).

**Table 2:** Comparison of various motor and sensory NCS parameters of lower limb between diabetic patients and controls Independent Student’s t-test; df = 58 for all comparisons NCS: nerve conduction studies; CMAP: compound muscle action potential; SNAP: sensory nerve action potential; HS: highly significant (p < 0.001)

Parameter	Nerve	Diabetics (n = 30), mean ± SD	Controls (n = 30), mean ± SD	Test statistic (t)	p-value	Significance
Distal Motor Latency (ms)	Common Peroneal	7.56 ± 1.48	3.23 ± 1.21	12.41	<0.001	HS
	Posterior Tibial	7.61 ± 1.32	3.61 ± 1.16	12.47	<0.001	HS
CMAP Amplitude (mV)	Common Peroneal	3.18 ± 1.05	7.61 ± 1.66	12.35	<0.001	HS
	Posterior Tibial	3.34 ± 1.08	7.89 ± 1.32	14.61	<0.001	HS
Motor Conduction Velocity (m/s)	Common Peroneal	33.01 ± 5.13	49.61 ± 4.66	13.12	<0.001	HS
	Posterior Tibial	32.71 ± 5.06	50.12 ± 5.02	13.38	<0.001	HS
F-wave Minimum Latency (ms)	Common Peroneal	66.87 ± 3.76	48.63 ± 3.95	18.32	<0.001	HS
	Posterior Tibial	67.23 ± 3.57	49.11 ± 3.56	19.69	<0.001	HS
Distal Sensory Latency (ms)	Sural	8.93 ± 1.93	3.23 ± 1.62	12.39	<0.001	HS
SNAP Amplitude (µV)	Sural	5.15 ± 1.88	8.21 ± 1.66	6.68	<0.001	HS
Sensory Conduction Velocity (m/s)	Sural	26.83 ± 3.21	41.61 ± 3.51	17.02	<0.001	HS

Correlation of NCS parameters with HbA1c

Higher HbA1c levels were associated with prolonged DML and DSL, higher F-wave minimum latencies, reduced CMAP and SNAP amplitudes, and slower MCV and SCV across all studied nerves. Most correlations were of weak to moderate strength, but statistically significant, and the corresponding t-statistics were consistent with the reported p-values. These results support a negative effect of poorer glycemic control on both myelin-related and axonal indices of nerve function (Table 3).

**Table 3:** Correlation of HbA1c with motor and sensory NCS parameters (diabetic group, n = 30) Pearson correlation; test statistic (t) calculated with df = 28 NCS: nerve conduction studies; HbA1c: glycated hemoglobin; CMAP: compound muscle action potential; SNAP: sensory nerve action potential

Parameter	Nerve	r-value	Test statistic (t)	p-value	Significance
Distal Motor Latency	Median	0.522	3.24	0.003	Significant
	Radial	0.487	2.95	0.006	Significant
	Ulnar	0.382	2.19	0.037	Significant
	Common Peroneal	0.365	2.07	0.047	Significant
	Posterior Tibial	0.332	1.86	0.062	Not significant
CMAP Amplitude	Median	-0.557	3.55	<0.001	Highly significant
	Radial	-0.459	2.73	0.010	Significant
	Ulnar	-0.568	3.65	<0.001	Highly significant
	Common Peroneal	-0.601	3.98	<0.001	Highly significant
	Posterior Tibial	-0.455	2.70	0.010	Significant
Motor Conduction Velocity	Median	-0.429	2.51	0.018	Significant
	Radial	-0.431	2.53	0.017	Significant
	Ulnar	-0.411	2.39	0.023	Significant
	Common Peroneal	-0.390	2.24	0.030	Significant
	Posterior Tibial	-0.378	2.16	0.0398	Significant (as per original table)
F-wave Minimum Latency	Median	0.656	4.61	<0.001	Highly significant
	Radial	0.597	3.94	<0.001	Highly significant
	Ulnar	0.615	4.13	<0.001	Highly significant
	Common Peroneal	0.646	4.48	<0.001	Highly significant
	Posterior Tibial	0.63	4.29	<0.001	Highly significant
Distal Sensory Latency	Median	0.579	3.76	<0.001	Highly significant
	Radial	0.602	3.99	<0.001	Highly significant
	Ulnar	0.521	3.23	0.003	Significant
	Sural	0.552	3.50	0.002	Significant
SNAP Amplitude	Median	-0.583	3.80	<0.001	Highly significant
	Radial	-0.598	3.95	<0.001	Highly significant
	Ulnar	-0.619	4.17	<0.001	Highly significant
	Sural	-0.609	4.06	<0.001	Highly significant
Sensory Conduction Velocity	Median	-0.619	4.17	<0.001	Highly significant
	Radial	-0.604	4.01	<0.001	Highly significant
	Ulnar	-0.627	4.26	<0.001	Highly significant
	Sural	-0.667	4.74	<0.001	Highly significant

Correlation of NCS parameters with age

Increasing age was associated with longer DML, DSL, and F-wave latencies, reduced CMAP and SNAP amplitudes, and slower MCV and SCV in both upper- and lower-limb nerves. Many of these correlations were moderate in magnitude and highly significant. The strongest age-related effects were observed for F-wave latencies, sensory latencies, SNAP amplitudes, and SCV, suggesting that aging exerts a measurable additive burden on sensory and proximal motor conduction in T2DM (Table 4).

**Table 4:** Correlation of age with motor and sensory NCS parameters (diabetic group, n = 30) Pearson correlation; test statistic (t) calculated with df = 28 NCS: nerve conduction studies; CMAP: compound muscle action potential; SNAP: sensory nerve action potential

Parameter	Nerve	r-value	Test statistic (t)	p-value	Significance
Distal Motor Latency	Median	0.641	4.42	<0.001	Highly significant
	Radial	0.648	4.50	<0.001	Highly significant
	Ulnar	0.691	5.06	<0.001	Highly significant
	Common Peroneal	0.539	3.39	0.002	Significant
	Posterior Tibial	0.431	2.53	0.017	Significant
CMAP Amplitude	Median	-0.414	2.41	0.023	Significant
	Radial	-0.381	2.18	0.038	Significant
	Ulnar	-0.287	1.59	0.182	Not significant
	Common Peroneal	-0.392	2.25	0.032	Significant
	Posterior Tibial	-0.327	1.83	0.078	Not significant
Motor Conduction Velocity	Median	-0.367	2.09	0.045	Significant
	Radial	-0.397	2.29	0.030	Significant
	Ulnar	-0.412	2.39	0.023	Significant
	Common Peroneal	-0.432	2.53	0.017	Significant
	Posterior Tibial	-0.442	2.61	0.014	Significant
F-wave Minimum Latency	Median	0.412	2.39	0.023	Significant
	Radial	0.400	2.31	0.029	Significant
	Ulnar	0.443	2.61	0.014	Significant
	Common Peroneal	0.346	1.95	0.060	Not significant (trend)
	Posterior Tibial	0.372	2.12	0.042	Significant
Distal Sensory Latency	Median	0.408	2.36	0.025	Significant
	Radial	0.339	1.91	0.066	Not significant
	Ulnar	0.439	2.59	0.015	Significant
	Sural	0.456	2.71	0.011	Significant
SNAP Amplitude	Median	-0.427	2.50	0.019	Significant
	Radial	-0.478	2.88	0.008	Significant
	Ulnar	-0.418	2.43	0.022	Significant
	Sural	-0.434	2.55	0.017	Significant
Sensory Conduction Velocity	Median	-0.408	2.36	0.026	Significant
	Radial	-0.369	2.10	0.045	Significant
	Ulnar	-0.418	2.43	0.021	Significant
	Sural	-0.492	2.99	0.006	Significant

Correlation of NCS parameters with duration of diabetes

Longer duration of T2DM showed significant associations with prolonged motor and sensory latencies, reduced amplitudes, and slowed conduction velocities in multiple nerves. The most pronounced duration-related changes were evident in F-wave latencies, sensory latencies, SNAP amplitudes, and SCVs, reflecting the progressive nature of diabetic nerve injury over time. Although the mean disease duration in this cohort was relatively short (3.38 ± 2.29 years), the presence of statistically significant correlations indicates that subtle, but measurable, deterioration in nerve function occurs even early in the disease course (Table 5).

**Table 5:** Correlation of duration of diabetes with motor and sensory NCS parameters (diabetic group, n = 30) Pearson correlation; test statistic (t) calculated with df = 28 NCS: nerve conduction studies; CMAP: compound muscle action potential; SNAP: sensory nerve action potential

Parameter	Nerve	r-value	Test statistic (t)	p-value	Significance
Distal Motor Latency	Median	0.634	4.34	<0.001	Highly significant
	Radial	0.603	4.00	<0.001	Highly significant
	Ulnar	0.540	3.39	0.002	Significant
	Common Peroneal	0.511	3.15	0.004	Significant
	Posterior Tibial	0.412	2.39	0.023	Significant
CMAP Amplitude	Median	-0.585	3.82	<0.001	Highly significant
	Radial	-0.459	2.73	0.011	Significant
	Ulnar	-0.610	4.07	<0.001	Highly significant
	Common Peroneal	-0.610	4.07	<0.001	Highly significant
	Posterior Tibial	-0.467	2.79	0.009	Significant
Motor Conduction Velocity	Median	-0.497	3.03	0.005	Significant
	Radial	-0.526	3.27	0.003	Significant
	Ulnar	-0.500	3.06	0.005	Significant
	Common Peroneal	-0.469	2.81	0.009	Significant
	Posterior Tibial	-0.435	2.56	0.016	Significant
F-wave Minimum Latency	Median	0.767	5.93	<0.001	Highly significant
	Radial	0.729	5.64	<0.001	Highly significant
	Ulnar	0.716	5.43	<0.001	Highly significant
	Common Peroneal	0.733	5.70	<0.001	Highly significant
	Posterior Tibial	0.746	5.93	<0.001	Highly significant
Distal Sensory Latency	Median	0.739	5.80	<0.001	Highly significant
	Radial	0.743	5.87	<0.001	Highly significant
	Ulnar	0.702	5.22	<0.001	Highly significant
	Sural	0.687	5.00	<0.001	Highly significant
SNAP Amplitude	Median	-0.684	4.96	<0.001	Highly significant
	Radial	-0.703	5.23	<0.001	Highly significant
	Ulnar	-0.720	5.49	<0.001	Highly significant
	Sural	-0.722	5.52	<0.001	Highly significant
Sensory Conduction Velocity	Median	-0.723	5.54	<0.001	Highly significant
	Radial	-0.727	5.60	<0.001	Highly significant
	Ulnar	-0.734	5.72	<0.001	Highly significant
	Sural	-0.732	5.69	<0.001	Highly significant

Correlation of NCS parameters with BMI

No statistically significant correlations were observed between BMI and motor or sensory nerve conduction indices in this study. The correlation coefficients were small in magnitude, and all corresponding t-statistics and p-values indicated a lack of meaningful association (Table 6).

**Table 6:** Correlation of BMI with motor and sensory NCS parameters (diabetic group, n = 30) Pearson correlation; test statistic (t) calculated with df = 28 NCS: nerve conduction studies; BMI: body mass index; CMAP: compound muscle action potential; SNAP: sensory nerve action potential

Parameter	Nerve	r-value	Test statistic (t)	p-value	Significance
Distal Motor Latency	Median	0.206	1.11	0.273	Not significant
	Radial	0.140	0.75	0.458	Not significant
	Ulnar	0.138	0.74	0.464	Not significant
	Common Peroneal	0.091	0.48	0.629	Not significant
	Posterior Tibial	0.093	0.49	0.616	Not significant
CMAP Amplitude	Median	-0.079	0.42	0.676	Not significant
	Radial	-0.111	0.59	0.558	Not significant
	Ulnar	0.048	0.25	0.799	Not significant
	Common Peroneal	-0.155	0.83	0.412	Not significant
	Posterior Tibial	-0.075	0.40	0.690	Not significant
Motor Conduction Velocity	Median	-0.226	1.23	0.228	Not significant
	Radial	-0.214	1.16	0.255	Not significant
	Ulnar	-0.206	1.11	0.274	Not significant
	Common Peroneal	-0.261	1.43	0.163	Not significant
	Posterior Tibial	-0.245	1.34	0.192	Not significant
F-wave Minimum Latency	Median	0.255	1.40	0.173	Not significant
	Radial	0.259	1.42	0.167	Not significant
	Ulnar	0.260	1.42	0.165	Not significant
	Common Peroneal	0.304	1.69	0.102	Not significant
	Posterior Tibial	0.277	1.53	0.138	Not significant
Distal Sensory Latency	Median	0.324	1.81	0.080	Not significant
	Radial	0.284	1.57	0.128	Not significant
	Ulnar	0.291	1.61	0.119	Not significant
	Sural	0.306	1.70	0.100	Not significant
SNAP Amplitude	Median	-0.182	0.98	0.335	Not significant
	Radial	-0.220	1.19	0.242	Not significant
	Ulnar	-0.165	0.89	0.382	Not significant
	Sural	-0.245	1.34	0.193	Not significant
Sensory Conduction Velocity	Median	-0.129	0.69	0.498	Not significant
	Radial	-0.165	0.89	0.384	Not significant
	Ulnar	-0.192	1.04	0.310	Not significant
	Sural	-0.215	1.16	0.253	Not significant

Summary of key clinical correlates

Overall, HbA1c, age, and duration of diabetes emerged as the factors most consistently associated with abnormal NCS parameters, whereas BMI did not demonstrate a significant relationship. Male gender also appeared to be associated with greater electrophysiological abnormalities, although this was not formally analyzed in a multivariable model in the present study.

## Discussion

This study suggests that neurologically asymptomatic T2DM patients may already exhibit significant abnormalities in both motor and sensory nerve conduction, compared with healthy controls. Similar findings from earlier studies indicate that diabetic neuropathy often begins subclinically, affecting both myelin and axonal components before clinical signs emerge [5-9,12-14,21-23].

Prolonged distal motor and sensory latencies, and reduced conduction velocities in our study, are suggestive of demyelination, while decreased CMAP and SNAP amplitudes may indirectly reflect axonal involvement, consistent with patterns reported in diabetic neuropathy research [5-9,21-23]. F-wave prolongation showed one of the largest between-group differences, indicating potential early proximal involvement, although this remains an electrophysiological inference rather than definitive mechanistic confirmation [10-12].

Poor glycemic control showed weak to moderate correlations with impaired nerve conduction in this cohort, aligning with previous evidence linking elevated HbA1c to microvascular and metabolic injury that contributes to conduction abnormalities [13-17,24-28]. Large-scale epidemiological studies, including UKPDS (United Kingdom Prospective Diabetes Study), support the broader association between chronic hyperglycemia and diabetes-related complications, such as neuropathy, although these findings do not directly confirm the associations observed in our study [28].

Age and duration of diabetes also showed significant associations with worsening nerve function, likely reflecting cumulative vascular and metabolic burden over time [7,16-18,21-23,26,27]. Even with relatively short disease duration in this cohort, measurable abnormalities were present, suggesting that neuropathic changes may begin early in T2DM [12,17]. However, these associations do not imply independent effects, as multivariate analysis was not performed.

BMI did not show significant correlations with any NCS parameters in this cohort, suggesting that adiposity alone may not influence nerve conduction in early disease stages [18]. However, this absence of association may reflect the modest sample size, and other metabolic or inflammatory factors could play more nuanced roles, requiring further study [24-27].

Overall, our findings support the role of NCS in identifying subclinical neuropathy in T2DM, and highlight the potential value of optimizing metabolic factors before the onset of disabling clinical features [5-9,24-28], although interventional benefits were not assessed in this study.

## Conclusions

Neurologically asymptomatic patients with T2DM showed measurable abnormalities in both sensory and motor nerve conduction parameters, compared with healthy controls, suggesting the presence of early subclinical neuropathic changes in this cohort. Poor glycemic control (higher HbA1c), older age, and longer duration of diabetes were notably associated with these electrophysiological changes, whereas BMI showed no clear relationship in this sample; however, these associations do not establish independent predictive value.

Routine consideration of NCS in high-risk or newly diagnosed T2DM patients may allow earlier detection of subclinical neuropathy and provide an opportunity to intensify glycemic and risk-factor management before the onset of overt symptoms and disabling complications.
